# Soil Organic Carbon Loss and Selective Transportation under Field Simulated Rainfall Events

**DOI:** 10.1371/journal.pone.0105927

**Published:** 2014-08-28

**Authors:** Xiaodong Nie, Zhongwu Li, Jinquan Huang, Bin Huang, Yan Zhang, Wenming Ma, Yanbiao Hu, Guangming Zeng

**Affiliations:** 1 College of Environmental Science and Engineering, Hunan University, Changsha, PR China; 2 Key Laboratory of Environmental Biology and Pollution Control (Hunan University), Ministry of Education, Changsha, PR China; 3 Department of Soil and Water Conservation, Yangtze River Scientific Research Institute, Wuhan, PR China; Centro de Investigacion Cientifica y Educacion Superior de Ensenada, Mexico

## Abstract

The study on the lateral movement of soil organic carbon (SOC) during soil erosion can improve the understanding of global carbon budget. Simulated rainfall experiments on small field plots were conducted to investigate the SOC lateral movement under different rainfall intensities and tillage practices. Two rainfall intensities (High intensity (HI) and Low intensity (LI)) and two tillage practices (No tillage (NT) and Conventional tillage (CT)) were maintained on three plots (2 m width × 5 m length): HI-NT, LI-NT and LI-CT. The rainfall lasted 60 minutes after the runoff generated, the sediment yield and runoff volume were measured and sampled at 6-min intervals. SOC concentration of sediment and runoff as well as the sediment particle size distribution were measured. The results showed that most of the eroded organic carbon (OC) was lost in form of sediment-bound organic carbon in all events. The amount of lost SOC in LI-NT event was 12.76 times greater than that in LI-CT event, whereas this measure in HI-NT event was 3.25 times greater than that in LI-NT event. These results suggest that conventional tillage as well as lower rainfall intensity can reduce the amount of lost SOC during short-term soil erosion. Meanwhile, the eroded sediment in all events was enriched in OC, and higher enrichment ratio of OC (ERoc) in sediment was observed in LI events than that in HI event, whereas similar ERoc curves were found in LI-CT and LI-NT events. Furthermore, significant correlations between ERoc and different size sediment particles were only observed in HI-NT event. This indicates that the enrichment of OC is dependent on the erosion process, and the specific enrichment mechanisms with respect to different erosion processes should be studied in future.

## Introduction

Soil erosion has attracted more and more attention from all over the world for its impact on carbon geochemical cycles between soils and the atmosphere [1], [2]. However, it is still a controversial issue on the role of soil erosion on carbon cycles, with the most famous debate is the carbon source or sink [3]–[7]. The substance of the issue is the poor understanding of soil erosion process and the included carbon dynamics. The Intergovernmental Panel on Climate Change (IPCC) [8] suggested that lateral carbon movement was the source of the greatest uncertainty in the global carbon balance. Furthermore, Kuhn et al. [1] indicated that the movement of soil organic carbon (SOC), both its particulate and dissolved forms, through agricultural landscapes is not fully understood.

Loss of SOC from the ecosystem occurs as a result of three processes: (i) physical removal by water (erosion); (ii) release of carbon into the atmosphere; and (iii) leaching [9], [10]. While in water erosion, most of the SOC is lost through the procedure (i). Researchers considered that the physical removal of SOC undergo four stages during the erosion processes [11]. Firstly, the macroaggregates are detached and dispersed into microaggregates by raindrop impact, and release organic carbon (OC) at the same time. Secondly, SOC is transported by runoff in form of either dissolved organic carbon (DOC) or sediment-bound organic carbon (SBOC). Thirdly, the coarse or heavy particles were deposited in micro-depression during the migration path. Eventually, the SOC with the transportable particles or runoff are transported to outlet and deposited in concave slopes and floodplains. However, these processes are related to a number of factors, namely, rainfall intensity and kinetic energy, infiltration and runoff rates, soil properties and soil surface conditions such as soil moisture, roughness, crop residues, slope length and steepness [12], [13]. Among them, rainfall intensity and tillage practice have become the focus of the erosion study. Lots of experiments were conducted to study the impact of rainfall intensity and tillage practice on soil delivery and nutrient loss [13]–[15].

However, most of the researches, under different rainfall intensities and tillage practices, focused on the SOC dynamics during erosion, were conducted in watershed [16]–[18] or laboratory [10], [13], [19]. Different points of view were observed between them for the variety of research conditions. For example, Lal et al. [20] suggested that no-till would decrease silt in rivers and lakes, which would lower transport of SOC and pollutant-laden sediments to aquatic ecosystems and reduce hypoxia. Also, some researchers indicated that conservation tillage practice reduce losses in soil and SOC [21], [22]. However, Cogle et al. [23] found that the lost carbon from 20 cm deep tillage was consistently less from zero tillage. In addition, the study scale is also considered to be an important factor impact on the movement of SOC [24]. Schiettecatte et al. [19] indicated that at a largerscale, due to the increased probability of sediment deposition by topography and vegetation, the sediment became more enriched in OC. The problem obtained within watershed scale is the representativeness of field conditions and the extent to which the data obtained with these microcosms can be extrapolated [25]. And for laboratory experiments, the experiment condition is too ideal to simulate the natural state. While simulated rainfalls at small plot scale had been applied to investigate the detachment and sediment transport capacity of runoff [26] and the effects of water erosion on soil properties and productivity [27], and important and meaningful results were obtained. So the study at plot scale in field condition is essential for improving the understanding of SOC dynamics under different erosion processes.

Therefore, field simulated rainfall events at small plot scale were performed in this study, and the objectives of present study were to: (i) examine the carbon lateral movement at plot scale in field runoff area, (ii) investigate the selective migration processes of SOC. Such information will be useful for the study of SOC transportation, also, provide basic data for SOC migration model.

## Materials and Methods

### 2.1. Ethics Statement

In this study, soil sampling and sample determinations conducted were permitted by the local authorities (i.e. Soil and Water Conservation Monitoring Station). We also obtained a permission from the local authorities for reporting research results to the public. In addition, the field studies did not involve endangered or protected species.

### 2.2. Study site

**Figure 1:**
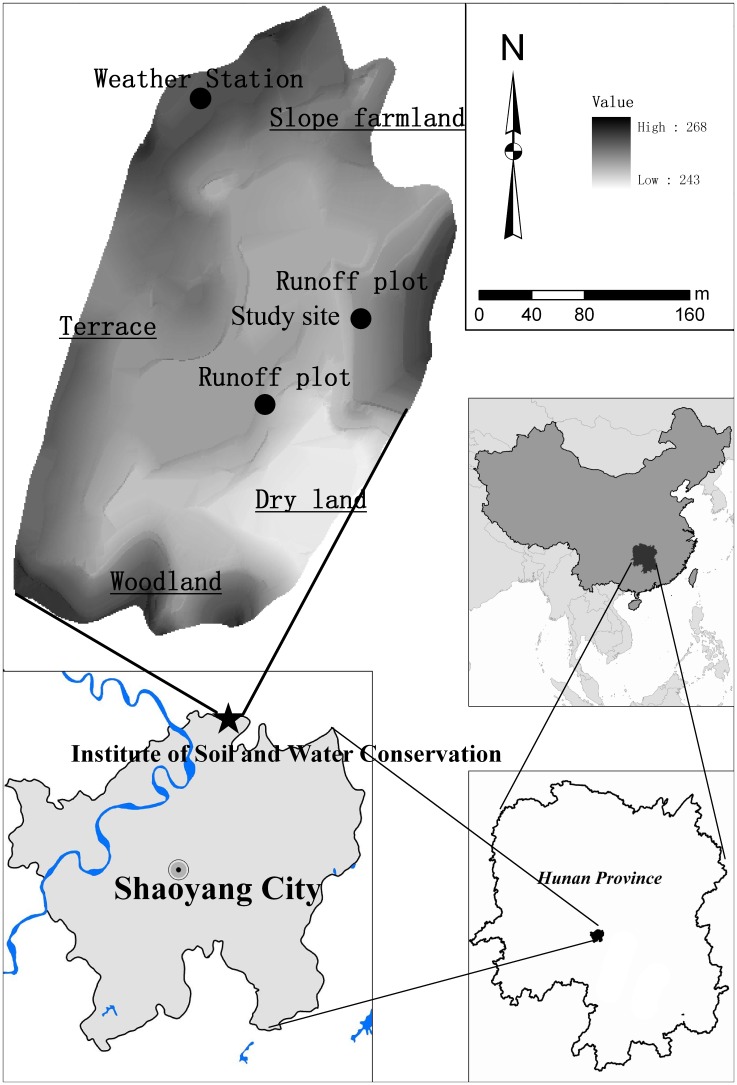
Location of the study area.

The simulated rainfall experiments were conducted at the Soil and Water Conservation Monitoring Station (111°22′ E, 27°03′ N), Hunan province, China (Fig. 1). The study area is located in subtropics humid monsoon climate zone, with an annual precipitation of 1 218.5 ∼1 473.5 mm and average annual temperature of 17.1°C. The record rainfall intensity of the last five years varied between 0.10 and 2.11 mm min^−1^, with 90% between 0.50 and 1.44 mm min^−1^. The area is characterized by hills and sloping lands with a gradient of 3 to 30%. The soil was developed from Quaternary red soil with sandy and clay loam texture. The area is a typical red soil hilly region. Due to the dense population and unreasonable land use, this region has suffered serious soil erosion, and more than 4 195.05 km^2^ cropland suffered water erosion with different degree. This area is representative of the agricultural, socio-economic and environmental situation of many slope farming areas in the region. The study carried out on this area is typical and representative of general situation in red soil hilly region.

### 2.3. Plot set-up and rainfall simulation

A 7 m×5 m block was taken from a typical sloping land with a 17% gradient to conduct the rainfall simulation. The block was previously planted with slope cultivated *Polygonatum odoratum* (Mill.) Druce. After harvesting the crops, this block was abandoned for almost one year, and it was almost bare before the experiments. The soil had a bulk density of 1.65±0.15 g cm^−3^, meanwhile with a water content of 0.15±0.02%. The soil pH was 4.47±0.10 (acidic soil), and the soil carbon is considered to be SOC. The mean SOC concentration of the surface layer was 7.47±2.48 g kg^−1^ dry soil (the mean value of 45 replicate samples ± standard error), the mean DOC concentration was 29.26±0.21 mg kg^−1^. The soil had a clay-loamy texture with 33.44±1.27% clay particle size distribution, 27.82±1.63% silt, and 38.73±1.74% sand. Before the experiments, the block was divided into three equal plots, and each plot was designed with 2 m (width) × 5 m (length), and named Plot I, II, III respectively. Two tillage practices were maintained on the three plots: plot I and II were applied no tillage (NT) and plot III was maintained conventional tillage (CT). The plot III was disk ploughed (10 cm), while others kept natural state. The three plots were separated with a 0.5 m wide space. Each plot was bound with a metal frame inserted into the ground 15 cm in order to prevent runoff from adjacent areas. To determine ERoc, plot soil was sampled in all plots at depth of 10 cm. The boreholes were later filled and carefully leveled in order to reduce the effects of soil sampling.

For the erosion experiments, a rainfall simulator with a SPRACO cone jet nozzle mounted on the top of fixed 4.57 m long stand pipes was built. The nozzles were placed on the boundary of the plots. The median drop size was 2.4 mm with a uniformity of 89.7%. According to the local rainfall intensity variation for the past five years, rainfall intensities of 0.4∼0.6 and 1.3∼1.5 mm min^−1^ were used, representing the low intensity (LI) and high intensity (HI) storms of this region. Plot I, II, III were treated with HI-NT, LI-NT and LI-CT, respectively. Calibrations of rainfall intensities were conducted prior to the experiments. Four simulators were used in HI event, and two were used in LI events. For the HI event, each simulator was located on the longer side and closed to the conner of the runoff plot, the two simulators in LI events were located on the similar positions and distributed in diagonal direction. Meanwhile, five rain gauges were used to measure the actual rainfall intensity, one was placed on the top of the plot, and four were distributed around the two sides. For each rainfall event, rainfall lasted 60 min after the overland surface runoff began. Once overland surface runoff began, random runoff samples were manually and intermittently collected at 6 min intervals using a 1000 mL kettle. Each collected sample were deposited, separated from the water, dried in a forced-air oven at 105°C until constant mass was achieved and weighed for the determination of sediment concentration. All other runoff and sediment samples were collected in a marked pail and the total runoff volume in 6 min was recorded. Another sample was taken from the thoroughly mixed pail, and this sample was splitted into two portions. One portion was dried in an oven at 105°C until constant mass and then weighed for the determination of physicochemical properties, the other portion was sieved with1, 0.5 and 0.25 mm pore openings for the separation of aggregates. The separated aggregates were dried and weighed separately. The actual rainfall intensity was determined after the simulated rainfall event through the rainfall gauges. The mean rainfall intensity was found to be 1.38 mm min^−1^ for the HI–NT event plot, 0.53 mm min^−1^ for the LI-NT and LI-CT event plot.

### 2.4. Sample treatment and data analysis

Soil bulk density was determined by cutting ring method. SOC concentrations of soil and sediment were determined with the dichromate oxidation method of Walkley and Black [28]. Soil particle sizes were analyzed using the pipette method [29]. Total organic carbon concentrations for the runoff samples were measured with a Shimadzu TOC-TN analyzer.

The ERoc of sediment was calculated by dividing the SOC content of the sediment by its content in the original soil material. In this study, the ERoc of sediment was the ratio between the SOC concentration of sediment and the value of the source soil for each plot.

### 2.5. Statistical analysis

Statistical data analysis was performed using SPSS 20.0 for Windows. Pearson correlation was used to test the significance of correlations among ERoc and sediment size as well as the sediment particle size distribution. Differences in correlation analysis were detected using the least significant difference procedure for a multiple range test at the 0.05 significance level.

## Results

### 3.1. Rainfall features

#### 3.1.1. Sediment and runoff loss

Through the trials, different sediment and runoff yield rates were distinguished. For the events of HI-NT and LI-NT, two stages of sediment and runoff loss were found. In the first stage, plot soil underwent infiltration and runoff starting, the rates of sediment and runoff loss rapidly increased in the initial rainfall time (0∼12 min). In the second stage, runoff loss rates reached steady state. The sediment loss rate in HI-NT event was consistent with the runoff loss rate. However, the values of sediment loss in LI-NT were first stable and then increased. In addition, different from these trends, the sediment and runoff yield rates in LI-CT increased consistently with the rainfall time.

The time to start runoff in LI-NT event was 1.66 times longer than that in HI-NT event, while, the figure in LI-CT event was 19.05 times longer than that in LI-NT event (Table 1). Despite longer time (additional 48 min) spent in runoff starting, the LI-CT event generated less runoff than LI-NT event did. The total runoff in HI-NT event was 2.43 times greater than that in LI-NT event which was 3.87 times higher than that in LI-CT event. Moreover, the sediments yield in HI-NT and LI-NT event was 68.34 and 14.38 times than that in LI-CT event, and which was correspond to the regular pattern of the lost runoff.

**Table 1:** The regular patterns of sediment and runoff transportation during water erosion.

	HI-NT (1.33 mm min^−1^), TSR: 1′31″				LI-NT (0.53 mm min^−1^), TSR: 2′31″				LI-CT (0.53 mm min^−1^), TSR:48′0″			
	SYR	CS	RR	CR	SYR	CS	RR	CR	SYR	CS	RR	CR
Sample number	(g min^−1^)	(g)	(L min^−1^ )	(L)	(g min^−1^)	(g)	(L min^−1^ )	(L)	(g min^−1^)	(g)	(L min^−1^ )	(L)
1	248.93	1493.58	5.62	33.70	45.33	271.99	1.62	9.70	1.25	7.48	0.42	2.50
2	815.48	6386.479	12.25	107.20	111.35	940.08	4.77	38.30	2.74	23.91	0.55	5.80
3	659.31	10342.35	11.87	178.40	92.08	1492.56	4.78	67.00	4.37	50.12	0.80	10.60
4	514.51	13429.40	12.08	250.90	99.52	2089.68	5.18	98.10	4.93	79.70	1.00	16.60
5	680.28	17511.10	11.67	320.90	94.67	2657.68	4.75	126.60	4.88	108.95	1.08	23.10
6	524.83	20660.10	13.22	400.20	92.05	3209.98	5.00	156.60	5.80	143.77	1.22	30.40
7	586.71	24180.35	12.88	477.50	119.98	3929.86	4.93	186.20	11.53	212.95	1.57	39.80
8	508.14	27229.17	11.48	546.40	161.07	4896.29	4.95	215.90	15.77	307.60	1.65	49.70
9	564.65	30617.05	11.18	613.50	212.03	6168.45	5.08	246.40	16.69	407.76	1.70	59.90
10	665.98	34612.93	12.37	687.70	171.08	7194.95	6.03	282.60	15.95	503.46	2.18	73.00

The first sample was collected at the time of runoff began, and samples were collected at 6 minutes intervals.

TSR, time to start runoff; SYR, sediment yield rate; CS, cumulative sediment yield; RR, runoff rate; CR, cumulative runoff volume.

#### 3.1.2. Sediment sorting

**Figure 2:**
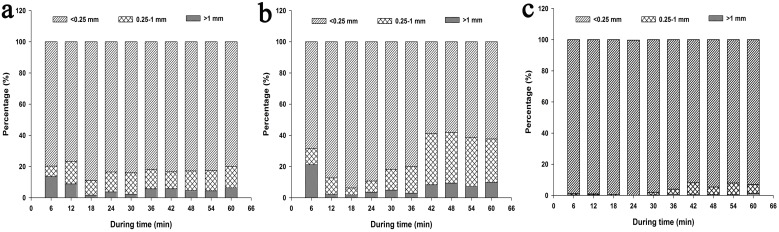
The distribution of different size aggregates in sediment in (a) HI-NT event, (b) LI-NT event, and (c) LI-CT event. HI-NT, high rainfall intensity-no tillage event; LI-NT, low rainfall intensity-no tillage event; LI-CT, low rainfall intensity– conventional tillage event.

For the soil aggregates, microaggregates can be preferentially transported and macroaggregates deposit easily during water erosion. In this experiment, the sediment was principally composed of <0.25 mm aggregate which accounted for more than 58% of the sediment yield (Fig. 2). The average proportion of 0.25–1 mm aggregate was lower than 20%, and the proportion of >1 mm aggregate was lower than 10%. For all the events, the proportions of aggregates in sediment decreased with increasing size. Further, the composition of aggregates varied with rainfall duration. For LI-NT and LI-CT events, the proportions of microaggregate (<0.25 mm) first increased and then decreased, eventually reached steady state. However, for HI-NT event, the proportions of aggregates were in stable state except in 6–18 min. The dynamics of aggregates also depend with rainfall events.

### 3.2. SOC loss and selective migration

#### 3.2.1. SOC loss

During water erosion, SOC loss in two forms: SBOC and DOC. Changes in loss rates of the SOC with respect to time were found to be very different among different rainfall events (Table 2). During the HI-NT event the loss rate of SBOC increased rapidly at the initial rainfall time, and then entered into a stable state. While For the LI-NT and LI-CT events, the loss rates of SBOC increased with the whole rainfall time. In comparison to SBOC, the loss rates of DOC presented various trends. For the HI-NT event, the DOC loss rate was relatively stable during the first 30 minutes and then decreased to the end. Meanwhile, the trends of fluctuation and increasing in DOC loss rates were found in LI-NT and LI-CT events, respectively.

**Table 2:** The regular patterns of SOC transportation during water erosion.

	HI-NT (1.33 mm min^−1^), TSR: 1′31″				LI-NT (0.53 mm min^−1^), TSR: 2′31″				LI-CT (0.53 mm min^−1^), TSR:48′0″			
	SBOCR	CSBOC	DOCR	CDOC	SBOCR	CSBOC	DOCR	CDOC	SBOCR	CSBOC	DOCR	CDOC
sample number	(g min^−1^)	(g)	(g min^−1^)	(g)	(g min^−1^)	(g)	(g min^−1^)	(g)	(g min^−1^)	(g)	(g min^−1^)	(g)
1	3.41	20.46	0.08	0.47	0.61	3.64	0.01	0.03	0.01	0.09	0.02	0.11
2	11.34	88.47	0.08	0.97	1.47	12.46	0.02	0.13	0.03	0.27	0.02	0.22
3	5.88	123.76	0.08	1.45	1.30	20.25	0.05	0.44	0.05	0.58	0.03	0.41
4	6.02	159.88	0.09	1.98	1.40	28.67	0.03	0.60	0.06	0.94	0.04	0.65
5	6.60	199.47	0.07	2.38	1.51	37.76	0.01	0.65	0.06	1.31	0.04	0.91
6	4.29	225.20	0.05	2.70	1.55	47.04	0.01	0.70	0.08	1.78	0.04	1.17
7	5.20	256.42	0.20	3.91	1.90	58.41	0.29	2.42	0.14	2.59	0.06	1.51
8	3.86	279.56	0.32	5.82	2.40	72.81	0.29	4.15	0.18	3.66	0.07	1.90
9	5.35	311.65	0.25	7.31	3.05	91.13	0.01	4.22	0.17	4.66	0.08	2.36
10	5.47	344.45	0.06	7.70	1.93	102.73	0.24	5.62	0.17	5.69	0.07	2.80

The first sample was collected at the time of runoff began, and samples were collected at 6 minutes intervals.

SBOCR, soil-bound organic carbon loss rate; CSBOC, cumulative soil-bound organic carbon; DOCR, dissolved organic carbon loss rate; CDOC, cumulative dissolved organic carbon.

The lost SOC (SBOC+DOC), SBOC and DOC in different events decreased in the order: HI-NT>LI-NT>LI-CT (Table 2). For NT plots, HI rainfall event had 3.25 times higher lost SOC than the LI events did. While for the LI events, NT plot had 12.76 times higher lost SOC than CT plot did, despite the CT plot had longer rainfall duration. Further, compared to the lost SOC, it was little and approximate for the DOC loss in each event. However, a considerable of lost SBOC under different rainfall events was observed. The values of SBOC/SOC reached 94% in HI-NT and LI-NT plot, the least was 67.02% in LI-CT. SBOC was the main form of the lost SOC.

#### 3.2.2. ERoc in sediment

**Figure 3:**
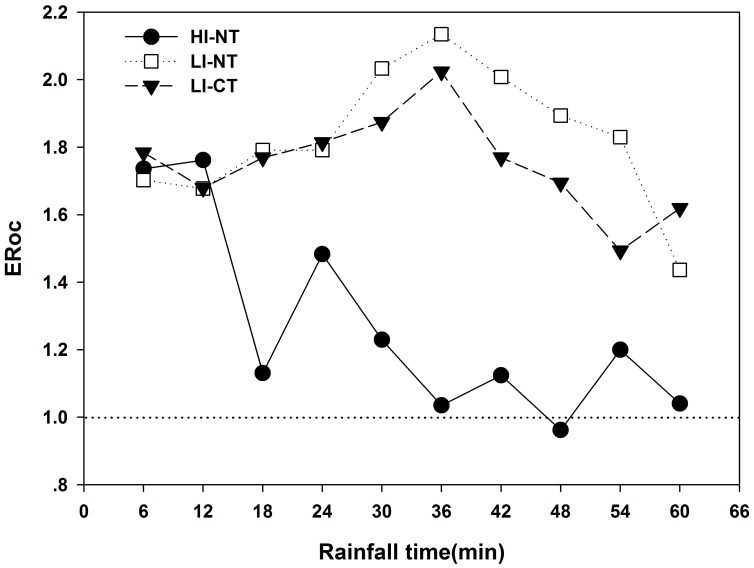
Dynamics of ERoc in sediment for different rainfall events. ERoc, enrichment ratio of organic carbon in sediment. HI-NT, high rainfall intensity-no tillage event; LI-NT, low rainfall intensity-no tillage event; LI-CT, low rainfall intensity-conventional tillage event.

In addition to the amount of the lost SOC, the selective migration processes were also studied. OC enrichment ratio (ERoc) in sediment for each event is presented in Fig. 3. ERoc curves for all the events were >1 except one value (0.96) in HI-NT event (48 min). For the events of LI-NT and LI-CT, ERoc curves had similar shapes, for example, the increasing stage (18–36 min), decline stage (36–54 min) and the peak value (36 min) were occurred at the same time. However, for HI-NT event, the ERoc curves decreased rapidly in fluctuation, and an exponential relationship (ER = 1.01+1.11 exp(−0.058t), R^2^ = 0.73) between ERoc and duration time was found. Under LI events, the tillage practices (NT and CT) had a moderate influence on ERoc, while in NT plots, the rainfall intensity (HI and LI) had a great impact on ERoc.

### 3.3. Correlation analysis

#### 3.3.1. The correlations of SOC and sediment and runoff

**Figure 4:**
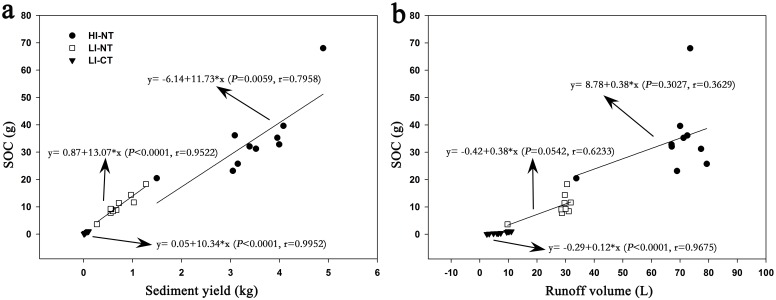
Correlations between the lost SOC and (a) sediment yield, (b) runoff volume. HI-NT, high rainfall intensity-no tillage event; LI-NT, low rainfall intensity-no tillage event; LI-CT, low rainfall intensity-conventional tillage event.

As the direct or indirect carrier of the lost SOC, runoff and sediment are important factors impact on the transportation of SOC. Fig. 4 displays the correlations between sediment yield, runoff volume and lost SOC for different rainfall events. Significant positive correlations (*P*<0.05) between sediment yield and lost SOC were observed in all rainfall events. First of all, the amount of the lost SOC increased with the increasing of the eroded sediment. Secondly, the linear correlations decreased with the increasing of the sediment yield (the correlation coefficient r decrease in the order: LI-CT>LI-NT>HI-NT). However, there was not significant linear relationship (*P*>0.05) between lost SOC and runoff volume for all the rainfall events except in LI-CT event. In LI-CT event, the runoff volume was very low, the lost SOC increased with runoff volume. While in LI-NT and HI-NT events, runoff increased rapidly, and finally got into a stable state.

#### 3.3.2. The correlations between ERoc and different size particles

Sediment particles transportation is often considered to be a cause of the selective migration of SOC. The correlations of sediment particles (different size aggregates (non-disperd particles) and sediment particle size distribution) and ERoc were analyzed (Table 3). The result shows that the correlations varied with rainfall events. In HI-NT event, ERoc had significant positive correlations with the content of clay (*P* = 0.011) and >1 mm aggregate (*P* = 0.042). Meanwhile, a significant negative correlation between ERoc and sand (*P = *0.041) was observed. However, for the events of LI-NT and LI-CT, there were not significant correlations between ERoc and soil particles, neither sediment aggregates nor sediment particle size distribution.

**Table 3:** Correlation analysis of ERoc and different size particles in sediment.

	Non-disperd particles			particle-size distribution		
ERoc	>1 mm	0.25–1 mm	<0.25 mm	sand	silt	clay
HI-NT	0.649*	−0.224	−0.523	−0.652*	0.236	0.760*
LI-NT	−0.343	0.105	−0.064	0.019	0.213	−0.088
LI-CT	−0.191	−0.468	0.462	–	–	–

Due to the small amount of sediment in LI-CT event, there were not enough samples to measure the particle-size distribution, and then default values were produced.

HI-NT, high rainfall intensity-no tillage event; LI-NT, low rainfall intensity-no tillage event; LI-CT, low rainfall intensity-conventional tillage event.

*Significant at 0.05 level.

## Discussion

### 4.1. SOC loss

The eroded carbon in all events was found to be mainly in form of SBOC (more than 67%, and as high as 90% in NT events). Similar results were obtained by Lowrance and Williams [30], who found that up to 90% of SOC in runoff may be in particulate phase. This result could be mainly explained by the distribution of different forms carbon in plot soil. In this study, the content of the DOC in original soil was 0.4% of the SOC, this means that most of the SOC is insoluble in water. Thus the original source of SBOC would be greatly guaranteed. Furthermore, the erosion intensity was also an important factor impact on the composition of the lost SOC. As showed in table 2, both the lost DOC and SBOC increased with erosion intensity, but the SBOC growth rate was much higher than DOC. This indicates that the more SOC lost, the higher proportion of SBOC in lost SOC.

The correlation analysis indicated that the lost SOC was significantly correlated to the eroded sediment (P<0.05), while not always correlated to runoff. This result showed that the lost SOC was more close to sediment, as well as the lost SOC was mainly in form of SBOC (Table 2), which indicated that sediment was the direct and main carrier of the lost SOC. While, runoff, as the limiting condition of sediment transport and detachment [26], did not affect the SOC transportation directly. This is consistent with the study result that nutrient loss during soil erosion was not directly related to runoff volume [23]. Consequently, it is appropriate to study the lost SOC through the study of sediment and sediment bound organic carbon.

Arnaez et al. [31] found that runoff increased linearly with rainfall intensity resulting in soil losses that also increased with rainfall intensity. Further, Zhang et al. [32] suggested that the amounts of eroded SOC were found to be strongly influenced by rainfall intensity. This study showed that more SOC was lost in HI-NT event in comparison to LI-NT and the amount of the lost SOC was significantly associated with sediment (*P*<0.05) (Fig. 4). In fact, the high rainfall intensity made seal formation and ponding time become shorter (Table 2), therefore, the infiltrated rainfall decreased quickly, meanwhile, runoff volume and velocity increased. In this way, rill erosion could become more intense and more soil and nutrients will be lost under high rainfall intensity event [13]. Consequently, higher rainfall intensity leads to great amount sediment eroded which result in more SOC loss.

While the amount of the lost SOC in LI-CT event can be ignored in comparison to that in LI-NT event. There are big difference between this result with the view that CT degrades soil structure and loosens soil surface which can accelerate soil erosion and SOC loss [21], [22], [33], [34]. Low amount of runoff and sediment yield were considered to be the reason of the negligible lost SOC in LI-CT event. Researchers suggested that under CT condition, the amount of macropores increases, infiltration is improved and the water storage capacity of soil becomes larger, also roughness of the field surface is reported to decrease runoff velocity [22], [35]–[37]. As a result, most of the rain water infiltrated to ground, and erosive power became very low. In general, CT changed the underlying surface properties and prevented soil loss. This result is consistent with that in the study of Cogle et al. [23], in which SOC loss in tillage areas was consistently less than that in NT areas. However, they considered that this benefit is of limited temporal value and not persistent. It is considered that the benefit occurred immediately post tillage, and before the soil had crusted [38]. Therefore, through the study, we believe that CT can make the underlying surface becomes rough and then reduce the loss of SOC, but this is limited by rainfall conditions. And we tend to attribute this result to the short rainfall time and low rainfall intensity used in our experiments. Therefore, more studies which were conducted with higher rainfall intensity and long-term monitor on CT plot should be taken in future. Nevertheless, the result is important for soil and water conservation in the areas which suffer from frequent short rains, for example the central southern China.

### 4.2. The selective transportation of SOC

Massey and Jackson [39] suggested that the ERoc often reflect the selectivity of OC. The study result showed that the ERoc values were >1 and indicated that the SOC could be transported preferentially. As the main carrier of the lost SOC, the eroded sediment also showed selectivity in migration process during erosion. The study result, the proportion of aggregates decreased with the increasing of aggregate size, indicated that the finer particles were preferential transported. Red soils in subtropical China are low in exchangeable sodium potential [40], and the main mechanisms of aggregate breakdown were by slaking due to fast wetting, mechanical breakdown due to raindrop impact and by runoff shear stress [41], [42]. With the impact of rainfall kinetic energy [43], stream power [42] on the aggregate breakdown and transportation, a sorting process in sediment (both sediment size and density) transport was found [42]. Due to nonhomogeneous distribution of nutrients among particles of various sizes and density [10], the selective migration of particles was considered to be one of the reasons of the enrichment of OC in sediment [44]. Martinez-Mena et al. [25] suggested that the selectivity has been partly attributed to the transported of fine-sized sediments which are richer in silt and clay particles. However, this study found that the correlations between ERoc and different size particles depend on rainfall events. Significant correlations between ERoc and sediment particles can be only found in HI-NT event. The large amount of the lost SOC (most of C was associate with the sediment particles) may help explain those significant correlations. While in LI-CT and LI-NT events, the ERoc had not any significant correlation with soil particles or sediment particle size distribution (Table 3). Jacinthe et al. [45] showed sediment collected during the low-intensity storms contained more mineralized carbon (30–40% of sediment carbon) than materials displaced during the high-intensity summer storms. And the preferentially transportation of poorly decomposed non-cohesive plant fragments are often attributed to the higher ERoc in sediment in low rainfall events [46].

Researchers suggested that sediment will become less enriched in carbon as time passes during an event since the more carbon-rich fine aggregates are depleted early in the event [25]. Whereas the increasing transport capacity of runoff is also considered to be related to the decreasing of ERoc [47], [48]. As a result, an exponential relationship in character (ERoc = 1.18+0.76exp(−0.046t), R^2^ = 0.81) between ERoc and rainfall duration (t) was found by Polyakov and Lal [10]. The same result of ERoc dynamics was observed in HI-NT event. However, for LI events, despite with increasing erosive power and sediment yield, an increasing trend of the ERoc was found during 12∼36 min. This suggests that the easiest transport substance were not the particles which have the highest OC concentration in LI events. Therefore, rainfall intensity had great impact on the ERoc in sediment and different enrichment mechanisms accounted for different ERoc dynamics. However, in contrast to rainfall intensity, tillage practices had a smaller impact on ERoc. For LI-NT and LI-CT events, ERoc curves had similar shapes. And the ERoc was almost the same in LI-CT and LI-NT plot when the erosive power of runoff was limited, while the difference could be observed when the erosive power growth at different degrees. Our results support the view that tillage affected the sediment yields but did not directly influence the ERoc [23]. Nevertheless, the impact of tillage on the ERoc mechanism is still unclear and further study is required.

## Conclusions

The regular patterns of SOC transportation during soil erosion processes were studied through field simulated rainfall experiments. These experiments showed that the LI-NT event had 12.76 times higher lost SOC than LI-CT event did. Conventional tillage can increase rainwater infiltration and reduce soil erosion and SOC loss. However, these results were obtained under short-term and low rainfall intensity event, and more experiments at long-term and storm events are needed to confirm these results. It was also found that SOC as well as sediment particles presented selective in erosion processes, and these processes were affected by rainfall intensity but not tillage practice. The selective transportation of finer particles was not always the reason for the enrichment of OC in sediment. The specific enrichment mechanisms of OC in sediment in relation to different erosion processes have to be studied in future. In addition, due to the complexity and uncertainties of field rainfall experiments, more similar field experiments should be carried out in the future.
